# Local Voices: Perspectives from the Local Community on the Primates of Kakamega Forest, Western Kenya

**DOI:** 10.3390/ani13223483

**Published:** 2023-11-11

**Authors:** Widava E. Ikhuluru, Malenya E. Imboma, Shikanga E. Liseche, Munayi J. Milemele, Sechero D. Shilabiga, Marina Cords

**Affiliations:** 1 Kakamega Monkey Project, Kakamega 50100, Kenya; erickwidava@gmail.com (W.E.I.); imbomaevans@yahoo.com (M.E.I.); shikangaernest@gmail.com (S.E.L.); jmunayi@gmail.com (M.J.M.); dero.shilabika@gmail.com (S.D.S.); 2 Department of Ecology, Evolution & Environmental Biology, Columbia University, New York, NY 10027, USA

**Keywords:** bushmeat, conservation, crop-foraging, ethnoprimatology, human perceptions, human–primate conflict, human–wildlife conflict, local knowledge

## Abstract

**Simple Summary:**

From the perspective of a field research team comprising members of the local community around a rain forest in western Kenya, we describe the relationship between local people and their local primates. The local community tends to have little knowledge about the natural history of the monkeys living nearby, and a negative attitude toward these animals which sometimes forage on maize, an important food crop. A minority of people value the meat of these monkeys, which they believe to enhance human health. However, attitudes are changing, along with people’s lifestyles. The local community today has less direct experience of the forest than previous generations, as more children go to school and more people find paid work. As a result of more widespread education, it appears that a more tolerant attitude, recognizing the role of wild primates in the forest ecosystem, is beginning to emerge.

**Abstract:**

From the perspective of a field research team comprising members of the local community near a rain forest in western Kenya, we describe the relationship between local people and local primates. Local people generally have little knowledge about the natural history of the monkeys living nearby, with people living closer to the forest knowing more. Most have a negative attitude toward monkeys because they occasionally forage on agricultural crops. A few people value monkey meat, which they believe to enhance human health. Participating in research on the behavioral ecology of blue monkeys allowed the author team to learn a great deal about these animals, including their role in the forest ecosystem and their behavioral similarities to humans. This experience differentiates their attitudes from most other members of their local community. However, the attitudes of local people are changing along with lifestyles. With more children in school and adults finding paid work, local people today generally have less experience of the forest than previous generations. A more tolerant attitude toward monkeys, recognizing their role in the forest ecosystem and their similarities to humans, is emerging among those who have been taught about biodiversity. This perspective is likely to contribute to their successful conservation.

## 1. Introduction

In a world dominated by human-caused environmental change, local people are necessarily a key part of conservation planning and action [1,2,3]. Understanding their needs, priorities, attitudes, and beliefs can help policy makers design effective strategies for wildlife conservation in a local context [4,5]. Yet a local community is not likely to be a homogeneous entity, and many rural communities are changing rapidly across the globe [6,7]. Recent studies have highlighted local variation in people’s knowledge of and attitudes toward their local primates [8,9,10,11,12]. It is with this variation in mind that we aimed here to describe local attitudes and beliefs about monkeys inhabiting a rain forest in western Kenya (Kakamega Forest) that is surrounded by a rural community living at high density. All but the last author of this work are part of this local community, and all authors have been part of a long-term research project focused on forest monkeys, especially blue monkeys (*Cercopithecus mitis stuhlmanni*; [13]), for 12–17 years (mean 14.4 years). We use our personal experience to describe local knowledge of the non-human primates (henceforth primates) of this forest, and to explore variation in the perspectives of members of the local community. We do this by addressing a series of questions on these topics, which we have discussed together as a group.

Kakamega Forest is an ‘island’ of rain forest in a ‘sea’ of densely human-populated farmland (>400/km^2^) in Kakamega East sub-county, Kakamega County, western Kenya [14]. The main forest block has a gazetted area of 238 km^2^ which includes non-forested habitats (e.g., grassy glades) as well as a mixture of forest types, including some that are heavily influenced by humans such as plantation forests of non-native trees (*Bischofia*, *Cupressus*, *Grevillea*, and *Pinus*), as well as more natural forest. The latter includes older (>50 years) mixed indigenous plantations and “near natural and old secondary forest” which experienced some commercial logging in the 1930-80s [15]. Many areas of the forest are used regularly by the forest-adjacent community as a source of fuelwood and building poles, with cattle grazing in some of the glades [16,17,18]. While Mitchell et al. [14] provide a useful summary, other important references about the forest’s composition and history include [15,16,19,20,21].

**Figure 1:**
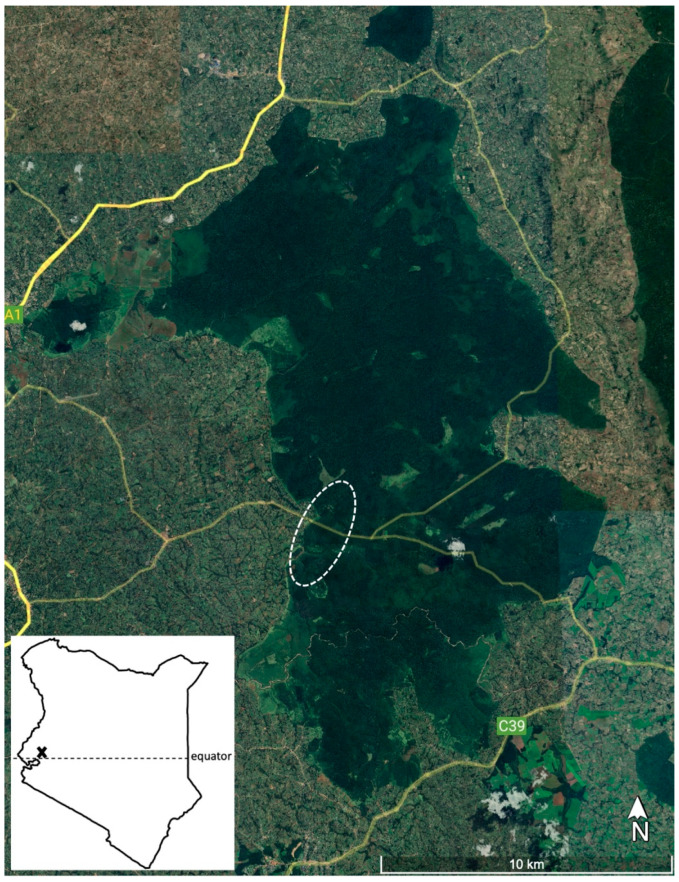
Image derived from Google Earth Pro showing Kakamega Forest, roads (yellow), and the study area (dashed white oval at 0°14′11″ N, 34°52′02″ E). The local community that our author set represents comes from the west/northwest of the study area. Inset map shows forest location (x) in Kenya (East Africa).

The Kakamega Forest ecosystem includes both the main forest block (Figure 1) and some outlying smaller islands of forest (Kisere, Malava, and Kaimosi). The main block is home to four species of diurnal primates, with black and white colobus (*Colobus guereza*), blue monkeys (*Cercopithecus mitis stuhlmanni*), and redtail monkeys (*C. ascanius schmidti*) being the most common, and olive baboons (*Papio anubis*) considerably rarer. Baboons are typically seen only in limited locations (there may be only one to two troops in the entire main forest block). Pottos (*Periodicticus potto*) are the only nocturnal primates.

Long-term research on blue monkeys has taken place in an area of ca. 2 km^2^ (dashed oval in Figure 1) surrounding the Kakamega Forest Station, located in the mid-western part of the forest (0°19′ N, 34°52′; [11]). This part of the forest is managed by the Kenya Forest Service, in contrast to the most northern region which falls under Kenya Wildlife Service supervision [18,22]. Our Kenyan co-authors come from the community inhabiting the area near the study site (Figure 1): all of them grew up in this area, no more than 5 km from the forest edge, and their families have inhabited this region for multiple generations.

We acknowledge from the start that our explorations may be particular to this region of the forest, because there is variation not only in how the forest has been managed and thus how the community relates to it (KFS vs. KWS, see above), but also in the human population around the forest perimeter. These people are members of the Luhya tribe (currently the second largest tribe in Kenya), but this ethnic group comprises multiple subtribes and clans. The Kenyan authors of this piece are all Isukhas (though they represent different clans therein), as are the majority of those living in the area of the forest near the study site.

We also acknowledge that the local knowledge we provide in this commentary is based on the personal experience and somewhat subjective knowledge of the authors. We did not interview other community members, and did not collect quantifiable data. The Kenyan authors comprise one woman and four men, ranging in age from 30 to 48 years (mean: 40.2). All have completed high-school, two also completed a post-secondary diploma course related to tourism and wildlife, and one of these two also completed an undergraduate degree in natural resources management. To gather the knowledge presented in this commentary, the co-authors met in pairs, trios, and as an entire group to discuss each of the questions in the next five sections.

## 2. What Do Local People Know about the Monkeys of the Kakamega Forest?

Basic knowledge about local primates is linked to how much experience people have with them, and thus largely to how far from the forest they live. The four diurnal species have distinct names in the local vernacular (*isialume* for blue monkeys, *ikhunga* for redtails, *induvili* for colobus, and *inguchi* for baboons), but people who are not directly familiar with these animals, i.e., those living at a distance of ca. 1 km or more, would probably group them all together simply as monkeys. Those living near the forest can generally recognize and distinguish by species some of the monkeys’ commonly heard loud calls (colobus ‘roar,’ blue monkey ‘pyow’), but other rarer calls (such as the redtail male’s ‘pop’ or ‘hack’) or calls that are less conspicuous (such as within-group chirps or grunts by the two guenons) are less likely to be distinguishable. Further, the functions of even the recognized calls are not likely to be understood.

Other details of the monkeys’ natural history, such as what they eat and their social lives, are also generally not well known, even by people living along the forest edge. For example, the fact that they live in groups with a family structure and a single resident male, and that these groups defend their territories against neighboring groups, is not likely to be appreciated, even by the forest-adjacent community. The one exception could be poachers, who have a reason to pay closer attention to the habits of the animals they hunt.

## 3. What Is the Attitude of People to the Monkeys of Kakamega Forest?

For a relatively small number of local people, monkeys provide a desirable form of meat, which is believed to be more nutritious than the meat of domestic animals, and even to have medicinal properties. For example, it is believed that eating monkey meat can help people with malaria or HIV, bolstering their immune systems (see [23] for a similar report from eastern Kenya). The medicinal quality of the meat is attributed to the monkeys’ consumption of forest plants, some of which are used by herbalists to treat human health conditions. These beliefs are also tied to a traditional view of diseases (and other problems) being spirits, each of which has its own animal or plant that acts as a repellant. Thus, monkeys as well as other wildlife have a “spirit niche”, and each is able to repel certain spirits. For example, blue monkeys are believed to be good for dealing with backbone problems in women. These beliefs are not widely held at present, but limited to members of families with a tradition of hunting. Such families often have no alternative livelihood options, as their members have not been to school; instead, they pick up hunting skills from their kin. Hunters are known by the wider community to have expertise about how eating different types of bushmeat can help with particular health problems. In some cases, local people turn to bushmeat because western medicine is inaccessible, requiring cash they do not have for transportation to health centers or for prescribed medications. In other cases, their health problems are ones that western medicine has failed to cure (so eating bushmeat is a “desperate remedy”), whereas others are believed to be invulnerable to western medicine, which might even make the problem worse. The wider community may therefore occasionally consider eating monkey meat, even if it is not a habitual practice.

Eating monkey meat can also play a role in identity for members of the community who have moved away. One of us knows an older, learned man who lives in Nairobi (the capital city), but returns to his home area near the forest on holidays. At that time, eating bushmeat is a way for him to “feel at home”, as this is not a food he could consume in the city where he spends most of his time. (His wife, from another tribe, will have none of it nor will his grown children. He even has his own cooking pot for this purpose.)

More commonly, local people have a negative attitude toward monkeys because they sometimes engage in crop-feeding. Even one animal (often an adult male) can do considerable damage in a short time. In this densely populated region, the loss of even a small portion of the staple maize crop angers farmers, many of whom are living an economically marginal existence, counting on their crops to feed their families.

The reaction of people to crop-foraging monkeys is nevertheless somewhat variable. Some will want to exact revenge, and will try to kill the perpetrator before it has left the farm, or to set traps along the boundaries of their fields, or inside the nearby forest. Others, however, are satisfied if the offending monkey is chased from their farm back to the forest. The latter may use other tactics too, like erecting scarecrows or actively guarding their fields during daylight hours when maize is ripe, to prevent recurrences of crop-raiding. Both adults and children (on weekends and school holidays) may participate in crop-guarding.

Several factors underlie these different responses to monkeys that have damaged crops. One is the degree to which people are interested in consuming bushmeat: killing a crop-foraging monkey provides a convenient “excuse” for putting such meat on the table, whether because of its potential medicinal value or because it comes at no monetary cost, in contrast to meat from a butchery.

A second reason for variation in responses to crop-feeding monkeys relates to the prevalence of Christianity in this region. The story of Noah’s Ark, and the recognition that all animals are “God’s creatures” (see also [24]) and even that monkeys are particularly human-like, leads some people to not pursue them. Such people recognize the monkeys’ right to live, and even the responsibility of humans to safeguard them. They may also fear punishment in the afterlife if they kill a monkey. We acknowledge that others have described Christianity, especially in comparison to eastern religions like Hinduism and Buddhism [25], as separating dominating humans from non-human animals [4]. This view has, however, also been questioned by some scholars, who highlight Biblical passages alluding to the importance of wildlife to God [26].

Our team has noticed that women are more likely to have this more tolerant attitude than men. Accordingly, it is women who more often call over a member of our team for advice when a monkey is on the farm, as they are looking for an alternative solution to killing it. Men, by contrast, are more likely to try to kill it. This gender difference suggests that empowering women is likely to lead to more primate-friendly solutions, especially as women typically spend more time with children and thus can pass along their more tolerant perspectives. Others have noted that environmental protection is a priority of local women in this region [27].

A third influence is knowledge about biological conservation. Those who have learned about the forest as an ecosystem, of which the primates are a part, may be more tolerant. Overall, however, it is probably a minority of people who are tolerant, because most are not yet educated about biological conservation. Most local people still see killing the monkey as an expedient way of protecting their crops from further damage.

## 4. What Are Some Traditional Beliefs about Monkeys?

Monkeys are viewed by many as clever, keen, and cheeky, and also aggressive in fighting back if harassed by humans. One of us was told as a child that their ancestors existed in living monkeys, probably as a way to scare the child and keep it from interacting with monkeys, who might scratch, bite, or steal food from its hand. 

Most people think that if they throw a stick at a monkey, it will grab it and throw it back at them. When monkeys feed and drop fruits and seeds, they are viewed as doing this on purpose, “throwing” seeds onto human observers. Another common belief is that monkeys—especially baboons and colobus—are less respectful toward women: they might try to attack a woman, but are more afraid of men. 

The loud roar choruses of colobus males are believed to have been a way to tell time before people had clocks or watches. These choruses, which sometimes occur in the dark of night, announced the coming daybreak.

Colobus skins are a traditional part of local ceremonies, including circumcision ceremonies. Any person who the community wishes to honor as a leader may also be dressed in these skins. Skins are normally kept by hunters (or may be purchased and kept by others), and rented out for such occasions. Some, however, will hunt animals for skins before ceremonies to avoid having to pay to rent them, even though killing wildlife is illegal. Colobus skins are favored over other monkeys because they are more variegated in color and thus more ornamental, and because the hair is longer.

A traditional belief is that eating the intestines of redtail monkeys helps women have smooth deliveries. Planting sweet potatoes along with some of the intestine is also believed to increase the size of the harvest.

## 5. Do Local People Appreciate the Role of Monkeys in a Larger Forest System?

We think this awareness is generally absent, unless the person has studied biology/ecology at school or has come to the forest as a visitor and been so informed by the tour-guides. Detailed knowledge of what monkeys eat—which would relate to seed dispersal or plant pruning—seems not to be present, and the concept of an ecosystem seems not to be part of common or traditional knowledge. This situation jives with a long historical legacy of colonial and Kenyan foresters avoiding systems thinking, considering the forest mainly as a source of timber. Local people, however, may recognize monkeys as attracting tourists and thus see them as part of an economic system, if not a biological one.

The role of monkeys in carrying diseases is not generally recognized either. Indeed, as noted above, the local community is more prone to seeing them (if eaten) as a way to reduce rather than increase disease load in people.

## 6. How Has Participating in Research Changed Our Own Attitudes about Monkeys?

In this section, “we” and “our” refers particularly to the Kenyan authors of this piece. Our attitudes about the monkeys differ a lot from most other local people, and have changed since we became part of the research team. For us, live monkeys became a resource, as we are part of a team that studies them. They provide us with a job, but they also give us learning experiences and inspiration. For most other people in our community, live monkeys are not a resource in these ways or any other way, and therefore their lives are not valued.

We have become aware that these animals play a key role in the forest ecosystem, dispersing certain seeds and enhancing germination rates, as undigested seeds pass through their gut, reducing their dormancy. Monkeys are part of the food web, also eating insects and occasionally vertebrates, and are themselves eaten by large raptors. We learned about food webs in school, but they became real to us as we spent time in the forest. Spending time with monkeys also helped us see the trees in the forest in a different way. When a tree is living, monkeys may eat its fruits, flowers, stems, or leaves, but when it dies, it provides different food—namely, insects, which are rich in protein. These changes helped us see that trees have value in the forest ecosystem, even when they are dead.

We realize that the different monkey species interact with each other in different ways. The blue monkeys sometimes chase redtails, but we have also seen these species groom each other. By contrast, relations between blue monkeys and black-and-white colobus are always antagonistic.

We have learned that the monkeys share many features with humans, and not only the obvious morphological ones, like their hands and eyes. Shared behavioral features include many aspects of their social lives: helping others to carry an infant, punishing another’s offspring, encouraging their own infant’s independence by refusing to share food during the weaning process, competing over food, reconciling after their fights, and forming long-lasting groups that energetically defend their territory and which comprise family units. These animals recognize one another’s calls, which seem to have specific meanings and trigger appropriate responses. They are also keen in identifying danger, and recognize the difference between friends and enemies: for example, they are well habituated to our research team, but wary around people they do not know. We also note their interest in fellow group members. When a monkey falls a long way to the ground, others often come to look and it seems that they are curious and concerned. We have sometimes become aware of an animal’s death because other group members were jointly attending to and vocalizing at the body in an unusual way: to us, that seems like mourning.

Knowing them as individuals brings further insights. We recognize that they have distinct personalities: some females are loners, some are always on the front line during intergroup aggression, some are caring mothers whereas others are not, some are bolder whereas others are shy. Males differ in popularity with the females, and some males kill infants when they take over a group, whereas others do not. Some females are particularly likely to sneak mating with males in neighboring groups. Individuals are ordered in a hierarchy, with high- and low-rankers. All these features also remind us of human behavior.

We sometimes even relate to these animals at a different level, given that we know their individual life stories. For example, one female in a study group disappeared abruptly in the last year, with circumstantial evidence suggesting she was snared, leaving her young infant behind. The infant began moving on the ground after losing its mother, and was eventually killed by a dog because it had no adult protection. This was sad!

We realize that when monkeys act “badly” from a human perspective, it is often because humans have unwittingly trained them to do so. The system of “shamba agriculture” has occurred alongside our study area [28]: it involves local people planting food crops for a few years in parts of the forest being replanted with young trees, until the new trees form a closed canopy, making agriculture impossible. This practice, while well intentioned to benefit the local community, introduces exotic food crops to wild monkeys in the surrounding forest, who previously had no exposure to these foods. Once they have tasted it, monkeys seem to value maize especially highly, and it is not surprising to us that they began raiding maize fields. If this system continues, it would be better not to allow the planting of maize, using beans and Irish potatoes instead. These crops are less attractive to monkeys, require less fertilizer, and mature more quickly.

We have other suggestions for mitigating conflict with monkeys on farms. First, cutting down trees in the forest reduces the supply of natural foods, and may drive these animals to search for food supplements elsewhere. Therefore keeping the natural environment intact is important in the long run. Scarecrows may keep monkeys from approaching a maize field. Guarding the field by tying dogs to the perimeter would likely be quite effective at keeping monkeys away, as they are very afraid of dogs. Using bells on ropes in the maize stalks to indicate disturbance and triggering rapid responses, posting human guards for a few weeks as the maize reaches maturity, and then cutting it promptly could also be advisable practices.

## 7. Conclusions

**Figure 2:**
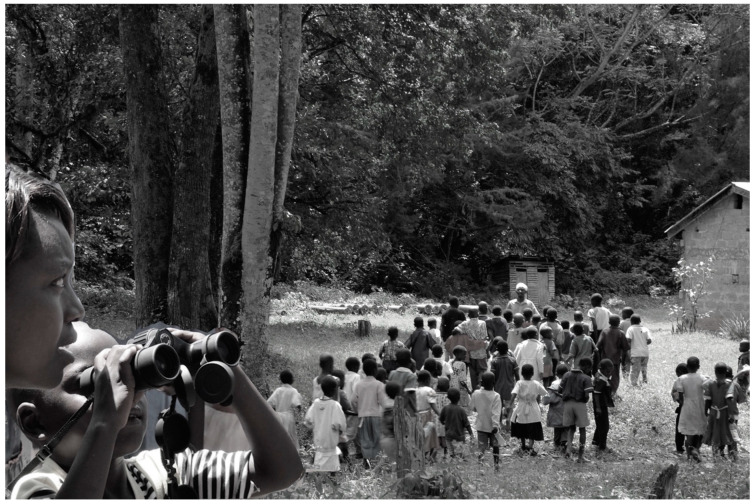
School children visiting the forest. Inset: under a teacher’s guidance, a child uses binoculars to see the local primates up close.

It is important in considering people’s attitudes toward local primates to recognize that attitudes are changing. Indeed, this makes our task here somewhat challenging. In our lifetimes, the habits and ambitions of people living in the forest-adjacent community (and throughout the country) have changed markedly. Today, it is relatively few people who have a close relationship to the forest, and the ongoing construction of an electric perimeter fence will reduce the number further. By contrast, in previous generations, the practices of gathering wood in the forest, taking cows to graze in its glades, or harvesting medicinal plants [29] meant that many, even most, people experienced the forest directly and often. They also viewed it as able to provide for their needs without limitation. Now, however, most children are going to school, which reduces their time in the forest; in addition, school learning displaces many traditional beliefs. In school, children learn that monkeys are related to humans as fellow primates, that the forest is a system of which the primates are a part, and that forest conservation has benefits for their community in terms of protecting ecosystem services. These taught ideas are changing people’s perceptions and attitudes. We see these changes evidenced in the busloads of schoolchildren that visit the forest (Figure 2). Teachers and local guides are primed to teach the pupils about the value of natural ecosystems and the benefits of their conservation. Some of us have also personally engaged in outreach with more local schools to spread this message. We believe these changes are good—and even necessary—for the persistence of the forest and the wildlife therein, and ultimately for the community living nearby.

## Data Availability

This commentary is not based on data; the narrative is effectively the data.
